# CD4^+^ T cells are required to improve the efficacy of CIK therapy in non-small cell lung cancer

**DOI:** 10.1038/s41419-022-04882-x

**Published:** 2022-05-06

**Authors:** Shaochuan Liu, Yuan Meng, Liang Liu, Yingge Lv, Wenwen Yu, Ting Liu, Limei Wang, Di Mu, Qiuru Zhou, Min Liu, Yulin Ren, Dong Zhang, Baihui Li, Qian Sun, Xiubao Ren

**Affiliations:** 1 Tianjin Medical University Cancer Institute and Hospital, National Clinical Research Center for Cancer, Tianjin, China; 2 Key Laboratory of Cancer Prevention and Therapy, Tianjin, China; 3 Tianjin’s Clinical Research Center for Cancer, Tianjin, China; 4 Key Laboratory of Cancer Immunology and Biotherapy, Tianjin, China; 5 Department of Immunology, Tianjin Medical University Cancer Institute and Hospital, Tianjin, China; 6 Department of Biotherapy, Tianjin Medical University Cancer Institute and Hospital, Tianjin, China

**Keywords:** Cancer immunotherapy, Immunoediting

## Abstract

As a widely studied adoptive treatment method, CIK (cytokine-induced killer cells) treatment has shown clinical benefits in many clinical trials on non-small cell lung cancer. As a heterogeneous cell population, however, CIK cells have a strong instability and individual differences in their efficacies, which are collaboratively regulated by the tumor microenvironment and CIK subpopulations. Among them, CD4^+^ T cells belong to a crucial subgroup of the CIK cell population, and their influence on CIK therapy is still unclear. Herein, we show how CD4^+^ T cells positively regulate the functions of CD3^+^CD56^+^ T and CD3^+^CD8^+^ T cells. During this process, we found that Th1/Th17 CD4^+^ subgroups can induce the phosphorylation of the AKT pathway by secreting IL-17A, and upregulate the expression of T-bet/Eomes transcription factors, thereby restoring the function of CD8^+^/CD3^+^CD56^+^ T cells and reversing the exhaustion of PD-1^+^Tim-3^+^ T cells. These findings will provide guidance for the clinical screening of suitable populations for CIK treatment and formulation of strategies for CIK therapy plus immune checkpoint treatment. Based on these findings, we are conducting an open-label phase II study (NCT04836728) is to evaluate the effects of autologous CIKs in combination with PD-1 inhibitor in the first-line treatment of IV NSCLC, and hope to observe patients’ benefits in this clinical trial.

## Abstract (web summary)

Herein, Liu and colleagues show that CD4^+^ T cells in cytokine-induced killer (CIK) cells are required to enhance the clinical efficacy of CIK therapy. In addition, CD4^+^ T cells help via IL-17A production is critical to restoring the function of CD8^+^/CD3^+^CD56^+^ T cells and reversing the exhaustion of PD-1^+^TIM-3^+^ T cells in CIK therapy.

## Graphical abstract

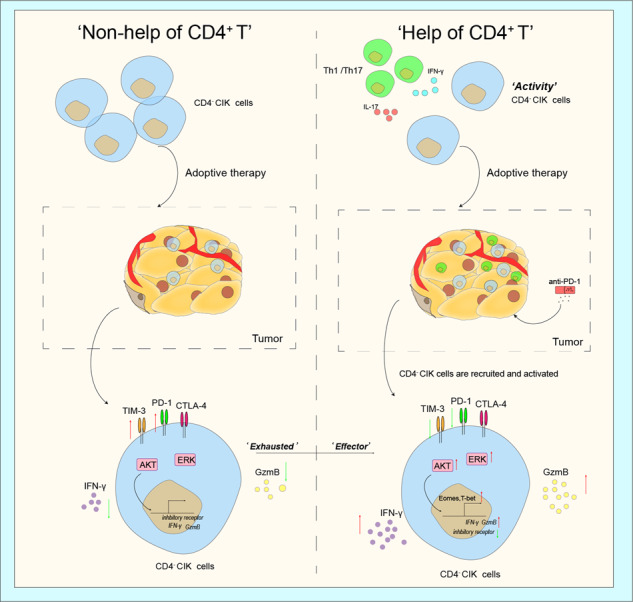

## Introduction

Cytokine-induced killer cells (CIKs), which are mainly CD3^+^CD56^+^ T cells, are a heterogeneous cell population obtained from human peripheral blood mononuclear cells (PBMCs) via ex vivo stimulation of multiple cytokines. These cells have a strong anti-tumor activity like the effector T cells and a potent MHC-unrestricted tumor-killing advantage like the NK cells [1]. CIK cell infusion, combined with DC (Dendritic cells), chemotherapy, or even immune checkpoint inhibitors are used clinically to treat various cancers and have obtained high clinical efficacy in lung cancer, hematologic tumor, and gastric cancer, etc. [2–6]. While CD3^+^CD56^+^ T cells are recognized as a crucial effector population of CIK cells against tumor cells, the purification of CD56^+^ cell does not significantly improve the capacity in killing tumor cells compared with CIK cells [7]. This suggests a close interaction between CD3^+^CD56^−^ T and CD3^+^CD56^+^ T cells, and CD3^+^CD56^−^ T cells may improve the killing capability of CD3^+^CD56^+^ T cells against tumor cells.

CD3^+^ T cells (more than 80%) predominate the CIK cell population, among which CD8^+^ T cells are the main population and the rest are CD4^+^ T cells. Most CD4^+^ T cells effectively improve both the memory and cytotoxic functions of CD8^+^ cytotoxic T lymphocytes (CTLs) and help CTLs to restrain tumor cells from immune escape through the release of multiple favorable cytokines in tumor microenvironment [8]. There is a significant difference in the proportion of CD4^+^ T cells in the CIKs from different patients through ex vivo expansion; in some patients, this proportion can even account for 60% of T cells. With a known critical role of CD4^+^ T cells in cancer immunity, we reasonably speculate that the different percentages of CD4^+^ T cells will ultimately determine the capacity of CIKs in killing tumor cells. We previously reported that Treg cells can inhibit the function of CIKs against tumor cells, especially by inhibiting the function of CD3^+^CD56^+^ T cells [9]. However, further investigation and exploration are needed to determine whether other CD4^+^ T cells subsets can increase or reduce the killing activity and cytotoxic function of CIKs. Hence, in vivo and in vitro experiments were designed to investigate the potential regulatory mechanism of CD4^+^ T cells in the CIK cell population for clinical screening of suitable NSCLC patients for CIK therapy.

## Results

### The percentage of CD4^+^ T cells correlated with clinical prognosis

**Fig. 1:**
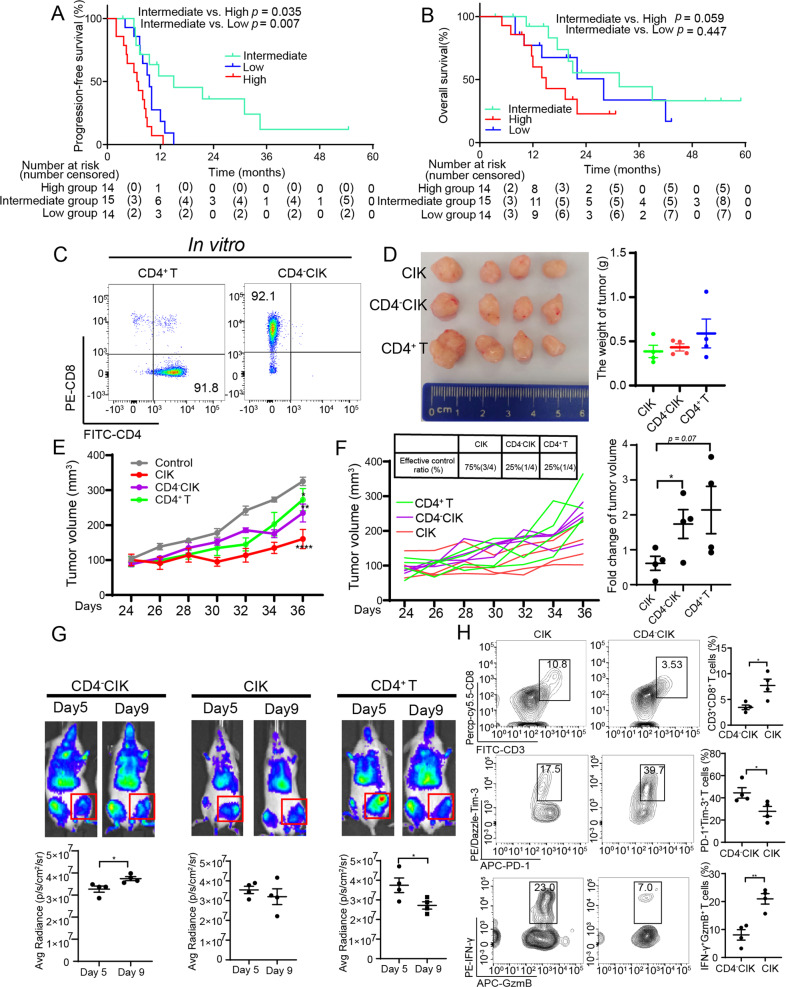
The percentage of CD4^+^ T cells correlated with clinical prognosis and function of CIK cells. **A** Kaplan–Meier estimates of progression-free survival in the three (high, intermediate, and low) groups. **B** Kaplan–Meier estimates of overall survival in the three (high, intermediate, and low) groups. **C** Flow cytometric quality assessment of CIK cells after 14 days of ex vivo expansion before adoptive transfer to tumor-burdened NSG mice. **D**–**F** Subcutaneous growth of tumor cells (A549) in each group of mice (*n* = 4) treated with CD4^+^ T, CD4^-^CIK, and CIK cells. **G** Bioluminescent imaging of NSG mice from three groups treated with T-cell tracer. **H** Flow cytometric analysis of IFN-γ, GzmB, PD-1, and Tim-3 expression in CD3^+^CD8^+^ T cells. Error bars indicate SEM, *P < 0.05, **P < 0.01, ***P < 0.001, and ****P < 0.0001 (Kaplan–Meier, one-way ANOVA or Student's *t* test).

We retrospectively collected the clinical information of patients from our previous clinical trial of NSCLC patients who received CIK therapy (NCT01631357) [10]. The baseline clinicopathological characteristics of the 43 patients are summarized in Supplementary Table S1. According to the percentage of CD4^+^ T cells in the CIKs population before they received the second CIK adoptive therapy, we divided these patients equally into high group (The mean value was 50.87%), intermediate group (37.77%), and low group (25.06%), and found that the intermediate group had a better progression-free survival (PFS) as compared to the other groups and a favorable trend for overall survival (OS) (Fig. 1A, B). Next, we further analyzed the correlation of a percentage of CD3^+^CD56^+^ T cells in the CIK cell population and prognosis, and found the high group (27.39%) to have a longer PFS than the low group (12.22%). In contrast, Treg high group (21.93%) has a poorer prognosis than the low group (8%) (Supplementary Fig. S1A, B).

In order to determine whether the percentage of CD4^+^ T cells was an independent prognosis factor for PFS, we performed a multivariate analysis and found the intermediate level (The mean percentages of CD4^+^ T cells and Treg cells were 37.77% and 10.92%, respectively.) to be a favorable prognostic factor for PFS; CD3^+^CD56^+^ T low group was an unfavorable prognostic factor for OS; only Treg low group was an advantageous prognostic factor for both OS and PFS (Supplementary Table S2). Based on the above research results, we put forward one reasonable assumption that CD4^+^ T cells had a significant role in CIK adoptive therapy.

### The number of CIKs was gradually increased over time at tumor sites

Before investigating the importance of the percentage of CD4^+^ T cells in CIK therapy, we need to understand the survival, tumor-killing ability, and movement of CIKs in vivo. Therefore, we expanded CIKs in vitro according to a previous study [11], and injected them intravenously into tumor-bearing NSG mice. We found that CIKs (mainly CD8^+^ and CD3^+^CD56^+^ T cells) could not only significantly inhibit the growth of tumors, but could also do not cause weight loss of mice and other graft–versus–host reaction (GVHR) (Supplementary Fig. S2A, B). Furthermore, the number and proportion of CD3^+^ T cells in various tissues (blood, spleen, liver, and lung) of mice gradually decreased over time, while the number and proportion of CD3^+^ and CD3^+^CD56^+^ T cells infiltration into tumor tissue gradually increased (Supplementary Fig. S2C–F). These results indicated that CIK cells could inhibit tumor cell growth in vivo, and also revealed the migration pattern of CIK cells to tumor tissues, which provided an experimental basis for the next animal experiments.

### CD4^+^ T cells were involved in regulating the recruitment and function of tumor-infiltrating CIKs

Based on the above results, to further investigate the impact of CD4^+^ T cells on CIK treatment, we used magnetic beads to enrich CD4^+^ T and CD4^-^CIKs respectively and detected 92.1% of the sorted CD4^-^CIK cells to be CD8^+^ T cells (Fig. 1C). We intravenously injected the enriched CIKs or CD4^+^ T cells into mice and observed that CD4^-^CIK and CD4^+^ T groups (25% of tumors effectively controlled) had a poorer ability to inhibit the growth of tumors than the CIK group (75%) (Fig. 1D–F). We performed in vivo imaging for each treatment group (stained with T-cell tracer) according to different time points and observed that CD4^-^CIK and CD4^+^ T-cell infiltration into the tumor had a different peak time, and the peak time of CD4^+^ T cells infiltration is earlier than that of CD4^-^CIKs infiltration. (Fig. 1G). This result suggests that different CIKs subgroups had the different migratory abilities, and there was a potential regulatory mechanism between the subgroups. In tumor tissues, we also found that the CD4^-^CIK group had a lower number of CD8^+^ T cells, a higher percentage of PD-1^+^Tim-3^+^CD8^+^ T cells (generally considered to be terminally exhausted T cells [12]), and a poorer cytotoxic function in tumor tissues than the CIK group (Fig. 1H). These results suggest that CD4^+^ T cells might be involved in regulating the recruitment and function of tumor-infiltrating CIKs.

### CD4^+^ T cells improved the migration of CD4^-^CIKs through IFN-γ/CXCL9,10,11/CXCR3

**Fig. 2:**
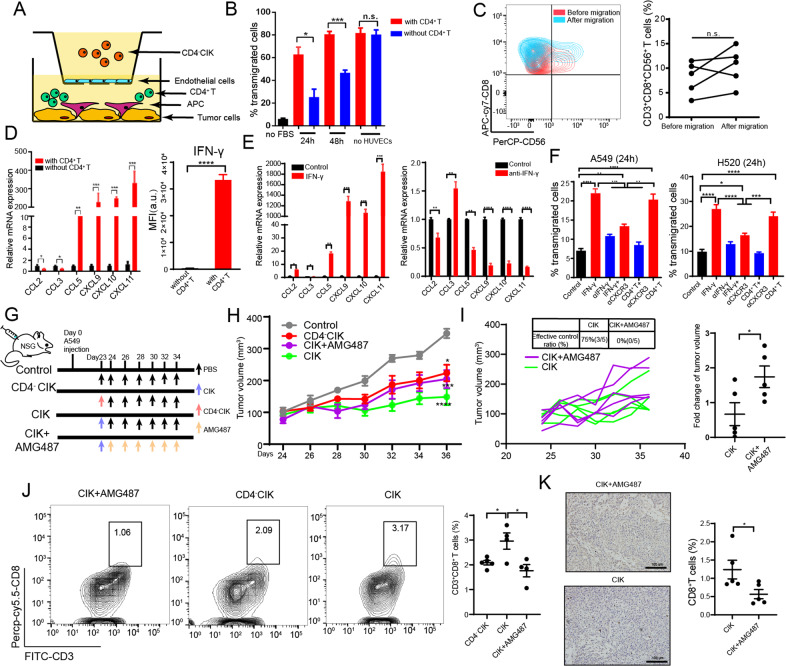
CD4^+^ T cells improved the migration of CD4^−^CIK cells through IFN-γ/CXCL9,10,11/CXCR3. **A** Illustration of chemotaxis assay of CD4^−^CIK cells toward supernatants derived from mixed tumor co-cultures. **B** Quantification of CD4^−^CIK cell migration in the tumor-conditioned medium in the presence or absence of CD4^+^ T cells. **C** Flow cytometric quantification of CD4^−^CIK migration in the tumor-conditioned medium before and after transmigration. **D** Quantification of chemokine-related mRNA expression in tumor cells and macrophages (left); flow cytometric analysis of IFN-γ expression in supernatants from tumor-conditioned medium (right). **E** Quantification of chemokine-related mRNA expression in tumor cells and macrophages in the presence of IFN-γ or anti-IFN-γ. **F** Quantification of CD4^-^CIK cell migration in tumor (A549 or H520)-conditioned medium in the presence of different neutralizing antibodies, IFN-γ or CD4^+^ T cells at 24 h. **G** Schematic diagram depicting the dosing schedule and timing for a subcutaneous transplantation tumor model. **H**, **I** Subcutaneous growth of tumor cells (A549) in each group of mice (*n* = 5) treated with PBS, CD4^-^CIK cells, CIK cells, and CIK + AMG487. **J** Flow cytometric examination of the number of CD3^+^CD8^+^ T cells infiltration in tumor. **K** Specific imaging of immunohistochemistry from CIK + AMG487 and CIK groups, scale bars: 100 µm. Error bars indicate SEM, *P < 0.05, **P < 0.01, ***P < 0.001, and ****P < 0.0001 (one-way ANOVA or Student's *t* test).

To assess cell motility and migratory potential of “CD4^+^ T cells-helped” CIKs versus “non-helped” CD4^-^CIKs, we performed in vitro transwell assays. We found that CIKs migrated significantly more efficiently through an endothelium than CD4-CIKs at both 24 h and 48 h (Fig. 2A, B). To examine whether the improvement for CD8^+^CD56^+^ T-cell migration is specific, we analyzed the percentage of CD8^+^CD56^+^ T cells in before and after migration of CIKs through the endothelial cell layer. We found no significant difference between before and after migration of the percentage of CD8^+^CD56^+^ T cells (Fig. 2C). Since effector T cells are known to infiltrate into tumor tissues under the guidance of inflammatory chemokines [13], we studied the mRNA expression of chemokines in tumor cells and macrophages in the co-culture system with CD4^+^ T cells and found that various chemokines (CXCL9-11) related to IFN-γ were significantly upregulated, and the mean fluorescence intensity (MFI) of IFN-γ was significantly increased in the supernatants (Fig. 2D). These results suggest that IFN-γ might play an important role in improving the migration of CD4^−^CIKs.

In order to confirm the importance of IFN-γ, we added IFN-γ recombinant protein or anti-IFN-γ antibody in the co-culture system and found that mRNA expression of CXCL9-11 were significantly increased in the IFN-γ stimulation and decreased with IFN-γ blockade (Fig. 2E). In the subsequent experiments, we also found that the number of CD4^-^CIKs migrating through the endothelium was reduced by the blockade of IFN-γ or CXCR3 (receptor for CXCL9, CXCL10, and CXCL11) (Fig. 2F). Next, we used AMG487 (anti-CXCR3 drug) to blockade the CXCR3 in vivo, and observed that the efficacy of CIK treatment was significantly inhibited (0% of tumors in CIK + AMG487 group was effectively controlled), exhausted T cells were significantly increased, and the number of tumor-infiltrating CD8^+^ and CD8^+^CD56^+^ T cells were also significantly reduced (Fig. 2G–K and Supplementary Fig. S3). These results indicate that CD4^+^ T cells improved the migration of CD4^-^CIKs through IFN-γ/CXCL9-11/CXCR3.

### CD4^+^ T cells enhanced the function of CIKs during ex vivo expansion

**Fig. 3:**
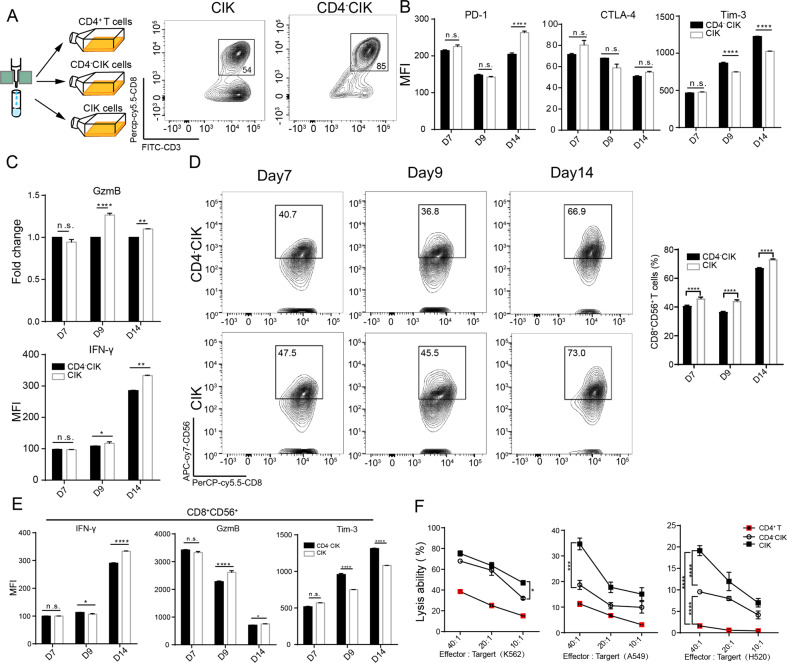
CD4^+^ T cells enhanced the function of CIK cells during ex vivo expansion. **A** Schematic diagram depicting the ex vivo expansion of different CIK cells (left); flow cytometric quality assessment of CIK cells when ex vivo expansion at day 14 (right). **B** Flow cytometric examination of immune checkpoint receptors expression in CD3^+^CD8^+^ T cells. **C** Flow cytometric analysis of IFN-γ and GzmB production in CD3^+^CD8^+^ T cells. **D** Flow cytometric examination of the percentage of CD3^+^CD8^+^CD56^+^ T cells in CD3^+^CD8^+^ T cells. **E** Flow cytometric analysis of IFN-γ, GzmB, and Tim-3 expression in CD8^+^CD56^+^ T cells. **F** The cytotoxicity of CIK, CD4^+^ T, or CD4^−^CIK cells against A549, H520, and K562. Error bars indicate SEM, *P < 0.05, **P < 0.01, ***P < 0.001, and ****P < 0.0001 (one-way ANOVA or Student's *t* test).

To evaluate the potential impact of CD4^+^ T cells on CD4^-^CIKs during ex vivo expansion, three groups of cells, namely CIK, CD4^+^ T, and CD4^−^CIKs, were collected, cultured and analyzed for their characteristics in vitro (Fig. 3A and Supplementary Fig. S4A, B). In CD4^−^CIK group, we found the Tim-3 expression on CD3^+^CD8^+^ T cells to be significantly up-regulated and secretions of cytokines (IFN-γ and granzyme B) to be evidently inhibited at day 14 (Fig. 3B, C). In addition, the number of CD8^+^CD56^+^ T cells was significantly reduced, Tim-3 expression was significantly increased and the potential secretory capacity of cytokines in CD8^+^CD56^+^ T cells was significantly weakened in the absence of CD4^+^ T cells (Fig. 3D, E). In subsequent killing experiments, we also found that CIKs had a more stronger killing ability compared to CD4^-^CIKs, for both K562, H520, and A549 (Fig. 3F). Next, to clarify whether the enhancement is dependent on intercellular contacts, we used a chamber (0.4 μm) and co-cultured CD4^+^ T cells with CD4^−^CIKs for 14 days (Supplementary Fig. S4C). We found that indirectly cultured CD4^-^CIKs did not differ significantly from normally cultured CIK in terms of function, inhibitory checkpoint expression, and killing capacity, while also significantly promoting CD56 expression on CD8^+^ T cells (Supplementary Fig. S4D–G). Taken together, these results suggest that CD4^+^ T cells promote the function of CIKs during ex vivo expansion and are not dependent on intercellular contacts.

### The supernatants of CD4^+^ T-cell culture improved the anti-tumor functions of CIKs in vitro

**Fig. 4:**
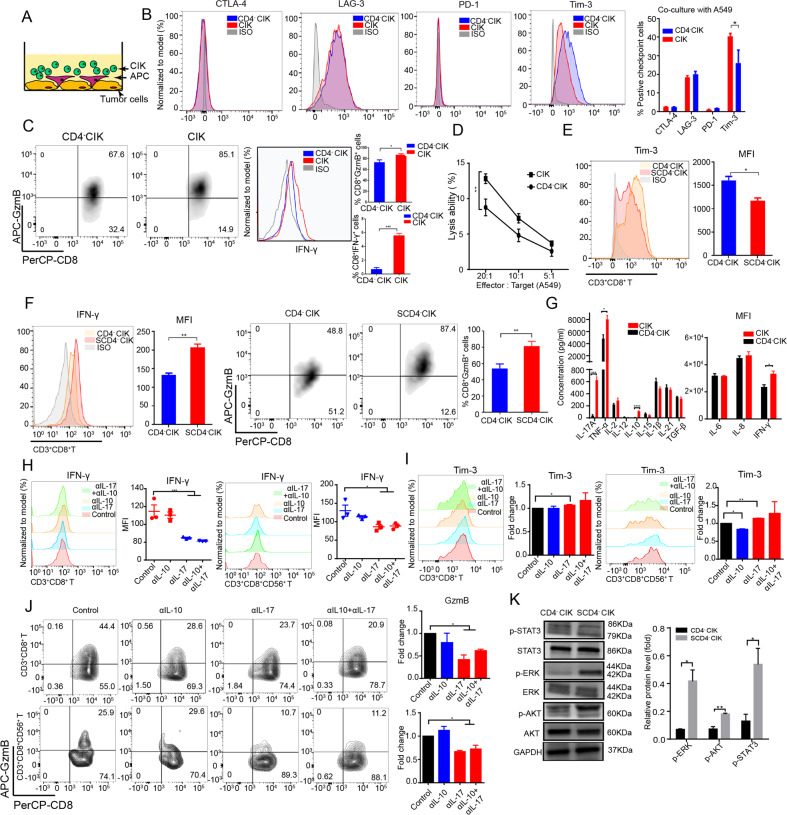
The supernatants of CD4^+^ T cell culture improved the functions of CIK cells in vitro. **A** Illustration of co-culture of tumor cells (A549 and H520) and CIK cells. **B** Flow cytometric analysis of PD-1, CTLA-4, Tim-3, and LAG-3 expression in CD3^+^CD8^+^ T cells in a co-culture system (A549). **C** Flow cytometric examination of GzmB and IFN-γ production in CD3^+^CD8^+^ T cells in a co-culture system (A549). **D** The cytotoxicity of CIK or CD4^−^CIK cells against A549. **E**, **F** Flow cytometric assessment of Tim-3, IFN-γ, and GzmB expression in CD3^+^CD8^+^ T cells treated with tumor-conditioned medium in the presence (SCD4^−^CIK group) or absence (CD4^−^CIK group) of CD4^+^ T cells. **G** Flow cytometric or ELISA assessment of the concentration or MFI of 12 human inflammatory cytokines in tumor-conditioned medium in the presence or absence of CD4^+^ T cells. **H**–**J** Flow cytometric analysis of Tim-3, IFN-γ, and GzmB expression in CD3^+^CD8^+^ T or CD3^+^CD8^+^CD56^+^ T cells treated with either PBS, anti-IL-10 (200 ng/ml), or anti-IL-17 (100 ng/ml). **K** Western blot analysis of STAT3, AKT, and ERK phosphorylation in CD4^−^CIK cells treated with tumor-conditioned medium for 2 h. Error bars indicate SEM, *P < 0.05, **P < 0.01, ***P < 0.001, and ****P < 0.0001 (one-way ANOVA or Student's *t* test).

To examine the effect of CD4^+^ T cells on CD4^-^CIKs in tumor environment, we made a model for co-culturing CIKs and tumor cells (A549 or H520) in vitro based on a previous study [14]. It is well known that T cells express a variety of inhibitory receptors in tumor microenvironment, which inhibit the activation and function of T cells [15]. Thus, we examined the inhibitory receptors on CIKs and found that CD3^+^CD8^+^ T cells had a higher Tim-3 expression, a poorer GzmB and IFN-γ release, and a poorer killing ability in CD4^-^CIK group compared to the CIK group. (Fig. 4A–D and Supplementary Fig. S5A). Tim-3 is widely known as a specific marker for the most dysfunctional CD8^+^ T cells during cancer progression [16]. Thus, we further analyzed Tim-3 expression on CD8^+^ T cells and found that the ability of CD8^+^ T cells to release IFN-γ and granzyme B (GzmB) was significantly inhibited with the elevated Tim-3 expression (Supplementary Fig. S5B). This result suggests that the high Tim-3 expression may imply CIKs dysfunction.

According to previous studies, CD4^+^ T cells could enhance the function of T cells by releasing various cytokines (such as IL-21 and IL-6) [17, 18]. Thus, we collected supernatants from the co-culture system containing CD4^+^ T cells and used them to stimulate CD4^-^CIKs. We found that CD4^−^CIKs stimulated by CD4^+^ T-cell supernatant (SCD4^**−**^CIK) had a stronger secretory capacity of cytokines and lower Tim-3 expression compared to the control group (Fig. 4E, F and Supplementary Fig. S6A).

To discern the cytokines involved in the improved function of CD4^-^CIKs, we analyzed the concentrations of the various cytokines and detected that the concentrations of IL-17A and IL-10 proteins in CIK group were higher than those in CD4^-^CIK group, except for effector factors IFN-γ and TNF-α (Fig. 4G). We used IL-17A and IL-10 neutralizing antibodies to block IL-17A and IL-10 proteins in the co-culture system, respectively. We found that Tim-3 expression on CD3^+^CD8^+^ T cells was significantly increased and cytokine secretions in CD3^+^CD8^+^ T cells were inhibited with IL-17A blockade; such phenomenon was not observed with IL-10 blockade alone (Fig. 4H–J). These results suggest that IL-17A could positively regulate the functional enhancement of CIKs in vitro. Previous studies have demonstrated that ERK, STAT3, and AKT pathways are involved in regulating the function of T and NK cells [19–22]. Thus, we further investigated the phosphorylation status of various pathways in CD4^−^CIKs, and found that SCD4^−^CIK group showed significant phosphorylation of ERK, STAT3, and AKT pathways compared with the control group (Fig. 4K). These results indicate that the supernatants of CD4^+^ T cells contributed to the activation of T cells through activating multiple phosphorylation pathways.

### CD4^+^ T cells improved the anti-tumor functions of CIKs through IL-17A

**Fig. 5:**
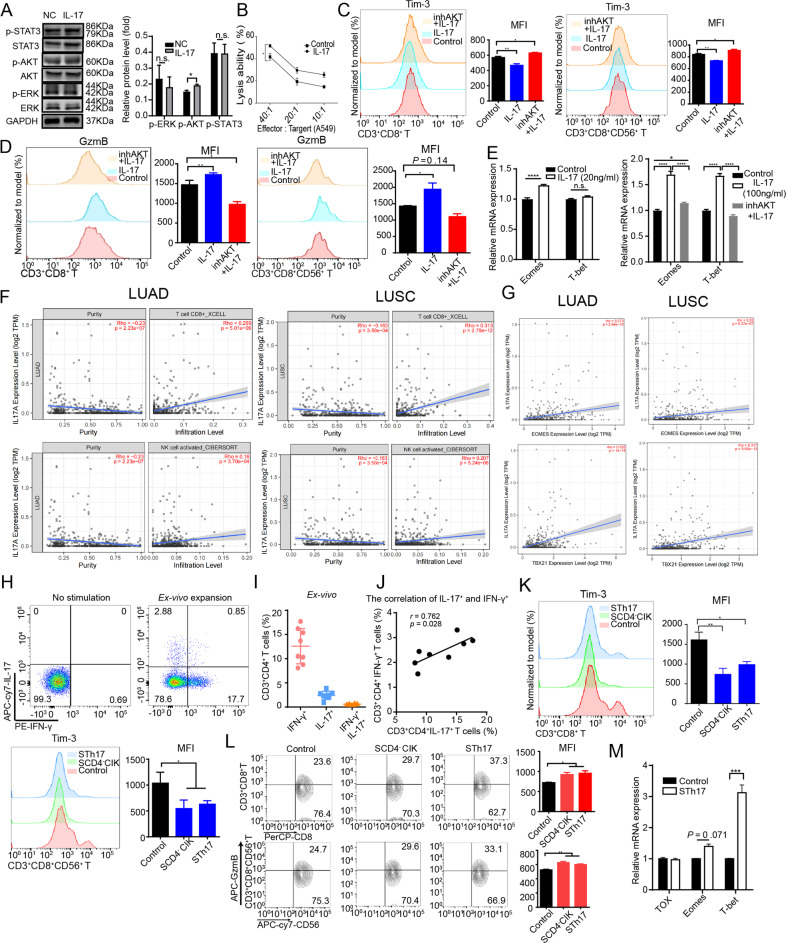
CD4^+^ T cells improved the functions of CIK cells through IL-17A. **A** Western blot analysis of STAT3, AKT, and ERK phosphorylation in CD4^-^CIK cells stimulated with rIL-17 (100 ng/ml) protein for 2 h. **B** The cytotoxicity of CD4^-^CIK cells treated with or without rIL-17 against A549. **C**, **D** Flow cytometric analysis of Tim-3 and GzmB expression in CD3^+^CD8^+^ T and CD3^+^CD8^+^CD56^+^ T cells treated with AKT inhibitor (Ly294002, 10 μM), rIL-17 or PBS. **E** Relative mRNA quantification of Eomes and T-bet in CD4^−^CIK cells treated with rIL-17, inhAKT, or PBS for 72 h. **F** Correlation analysis of IL-17A and CD8^+^ T cells infiltration or NK cells activated infiltration in LUAD and LUSC in TIMER public database. **G** Correlation analysis of IL-17A and *EOMES* or *TBX21* in LUAD and LUSC in TIMER public database. **H** Flow cytometric examination of IL-17 and IFN-γ production in CIK cells before and after ex vivo expansion. **I**, **J** The relationship of Th1 and Th17 cell groups (*n* = 8). **K** Flow cytometric analysis of Tim-3 expression in CD3^+^CD8^+^ T and CD3^+^CD8^+^CD56^+^ T cells treated with tumor-conditioned medium in the presence of Th17 cells, CD4^+^ T cells or absence of these cells. **L** Flow cytometric determination of GzmB production in CD3^+^CD8^+^ T and CD3^+^CD8^+^CD56^+^ T cells. **M** Relative mRNA quantification of Tox, Eomes, and T-bet in CD4^−^CIK cells treated with tumor-conditioned medium for 72 h. Error bars indicate SEM, *P < 0.05, **P < 0.01, ***P < 0.001, and ****P < 0.0001 (one-way ANOVA or Student's *t* test).

To further explore whether IL-17A improved the function of CIKs by these phosphorylation pathways, we used rIL-17A protein to stimulate CD4^−^CIKs and found that IL-17A could effectively induce the phosphorylation of AKT, enhance the secretion of GzmB, inhibit Tim-3 expression, and improve the cytotoxicity of CD4^-^CIKs (Fig. 5A–D). However, IL-17-mediated functional improvement was inhibited with AKT inhibitor (Ly294002) pretreatment (Fig. 5C, D). The PI3K-AKT pathway is known to enhance the proliferation of T cells and upregulate T-bet transcription [23, 24]. T-bet is a critical transcription factor for the cytotoxicity of NK cells, and promotes the transcription of genes including *Gzmb* and *IFNg* [19, 25]. Thus, we further investigated the relationship between IL-17 and transcription factors. We found that Eomes and T-bet mRNA expression were significantly increased under the stimulation of IL-17A (20 ng/ml or 100 ng/ml), but the upregulated mRNA expression also were inhibited by pretreatment of Ly294002 (Fig. 5E). We then analyzed the public database of Tumor IMmune Estimation Resource (TIMER2.0) and found that IL-17A was positively correlated with the number of activated NK and CD8^+^ T cell infiltration in LUAD and LUSC. Meanwhile, we also detected that IL-17A was positively correlated with *TBX21* and *EOMES* expression (Fig. 5F, G). Collectively, these results suggest that the PI3K–AKT–T-bet/Eomes axes were crucial regulators of the IL-17-mediated functional improvement of CIKs.

IL-17A is an important cytokine mainly produced from Th17 cells. To examine whether Th17 cells had the same effect in improving the function of CIKs, we examined the proportion of Th17 cells in CIKs. While Th17 cells occupied a certain proportion in the CIKs, we found that the CIK population is mainly Th1 cells (Fig. 5H, I). Furthermore, we found that there was a positive correlation between IFN-γ and IL-17A expression (Fig. 5J). Based on this point and the above results, we postulate that Th17 and Th1 work together to exert an anti-tumor effect in CIK therapy. Next, we cultured Th17 cells in vitro, and collected them supernatant from co-culture system to stimulate CD4^-^CIKs. We found that the supernatant of Th17 cells (STh17) could effectively activate CD4^-^CIKs, inhibit Tim-3 expression, enhance the secretory ability of GzmB, and upregulate T-bet expression (Fig. 5K–M). This result suggests that Th17 cells could effectively improve the function of CD4^-^CIKs by up-regulating T-bet expression.

### CIKs therapy plus IL-17A or anti-PD-1 treatment reversed the functions of exhausted CIKs

**Fig. 6:**
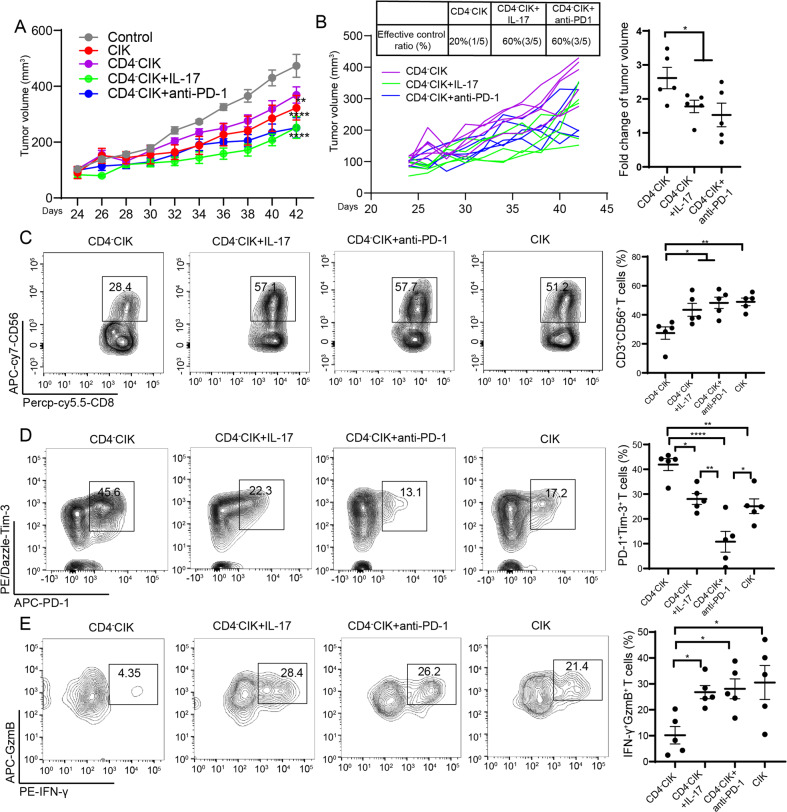
CIK cell therapy plus IL-17A or anti-PD-1 treatment reversed the functions of exhausted CIK cells in vivo. **A**, **B** Subcutaneous growth of tumor cells (A549) in each group of NSG mice treated with CD4^-^CIK cells (*n* = 5), CD4^-^CIK cells+anti-PD-1 (*n* = 5), CD4^-^CIK cells+rIl-17 (*n* = 5), or CIK cells (*n* = 5). **C** Flow cytometric examination of the percentage of CD3^+^CD8^+^CD56^+^ T cells in CD3^+^CD8^+^ T cells. **D** Flow cytometric analysis of the percentage of PD-1^+^Tim-3^+^CD8^+^ T cells in CD3^+^CD8^+^ T cells. **E** Flow cytometric determination of the percentage of GzmB^+^IFN-γ^+^CD8^+^ T cells in CD3^+^CD8^+^ T cells. Error bars indicate SEM, *P < 0.05, **P < 0.01, ***P < 0.001, and ****P < 0.0001 (one-way ANOVA or Student's *t* test).

To further confirm the positive regulatory relationship of IL-17A and function of CD4^-^CIKs, we performed an animal experiment. We found CD4^-^CIK + rIL-17A group (60% of tumors effectively controlled) significantly inhibited the growth of tumors compared to CD4^-^CIK group (20%) (Fig. 6A, B). Next, we analyzed the number, function, and exhaustion of CD4^-^CIKs infiltration, and found that the number of CD3^+^CD56^+^ T-cell infiltration was significantly increased, PD-1^+^Tim-3^+^ T cells were significantly decreased, and IFN-γ^+^GzmB^+^ T cells were significantly increased in CD4^-^CIK + rIL-17A group. These results suggest that the functional exhaustion of PD-1^+^Tim-3^+^ CIKs in tumor tissues could be restored by exogenous rIL-17A (Fig. 6C–E). In addition, the above clinical prognostic results already insinuate that patients with a low proportion of CD4^+^ T cells in the blood had a poor clinical efficacy in CIK therapy. In order to improve the therapeutic effect for these patients, combination therapy might be a feasible treatment strategy. Firstly, we added anti-PD-1 Abs or anti-Tim-3 Abs into the co-culture system and found that blockade of PD-1 could effectively improve cytokines secretion in CD8^+^ T cells, for both IFN-γ and GzmB. However, anti-Tim-3 only resulted in the upregulation of the GzmB secretion in CD8^+^ T cells (Supplementary Fig. S6B). This result indicates that anti-PD-1 therapy was more effective in promoting the function of CD4^-^CIKs compared to anti-Tim3 therapy. Based on this result, we performed animal experiments and found that CD4^-^CIK + anti-PD-1 (60% of tumors effectively controlled) groups significantly inhibited the growth of tumors (Fig. 6A, B). Moreover, we found that the number of CD3^+^CD56^+^ and IFN-γ^+^GzmB^+^ T cells infiltration were significantly increased, and PD-1^+^Tim-3^+^ T cells were significantly decreased in CD4^-^CIK + anti-PD-1 group (Fig. 6C–E). We further analyzed the function and exhaustion in CD3^+^CD8^+^CD56^+^ T cells, and found a similar phenomenon as well as CD3^+^CD8^+^ T cells (Supplementary Fig. S6C). Taken together, these results suggest that the functional exhaustion of PD-1^+^Tim-3^+^ CIKs could be restored by exogenous IL-17A or anti-PD-1 treatment.

## Methods

### Cell line generation and culture

Human lung adenocarcinoma cell line (A549), human umbilical vein endothelial cells (HUVECs), human lung squamous cell carcinoma cell line (H520), human lung adenocarcinoma cell line (H1299) and K562 (ATCC) were cultured in 1640, DMEM and IMEM supplemented with 10% FBS and 1% penicillin–streptomycin. All cell lines were obtained from ATCC and tested negative for mycoplasma contamination. CIKs were generated from ex vivo expansion of PBMC for 14 days as previously described [11]. CD4^−^CIK, CIK, and CD4^+^ T cells were cultured based on the established protocol of conventional CIKs culture and examined for IFN-γ, GzmB, PD-1, Tim-3, and CTLA-4 expression on day 7 or 14 by flow cytometry [11].

### Clinical data analysis

A total of 43 patients with advanced non-small-cell lung cancer (NSCLC) who were treated with CIK therapy in a randomized, multicenter, open-label clinical trial (ClinicalTrials. gov number, NCT01631357) were retrospectively reviewed. Inclusion criteria: the patient had received at least two or more CIK treatments. All clinical data, including age at diagnosis, gender, smoking history, TNM stage, and clinical stage, were recorded and analyzed by SPSS. The study was approved by the ethics committee of Tianjin Medical University Cancer Institute and Hospital, and informed consent was obtained from all subjects.

### Quantitative real-time (q)PCR

Total RNA was obtained from tumor cells, CIK cells, CD4^−^CIK cells, and macrophages with TRIzol reagent. The RNA extracted by chloroform was reverse-transcribed to cDNA using PrimeScript™ RT Master Mix (Perfect Real Time) for RT-PCR. Quantitative RT-PCR was performed using 2X SG Fast qPCR Master Mix (Low Rox) (Sangon Biotech) according to the manufacturer’s instructions. Each real-time PCR experiment included technical replicates, in a final volume of 20 µL. The sense and anti-sense primer sequences were shown as follows: CCL2 F: ACCAGCAGCAAGTGTCCCAAAG and CCL2 R: TTTGCTTGTCCAGGTGGTCCATG; CCL3 F: CTGGCTGCTCGTCTCAAAGTAGTC and CCL3 R: CATGGCTCTCTGCAACCAGTTCTC; CCL5 F: ACCCTGCTGCTTTGCCTACA and CCL5 R: ACTGCTGGGTTGGAGCACTT; CXCL9 F:TCATCTTGCTGGTTCTGATTGGAGTG and CXCL9 R: GATAGTCCCTTGGTTGGTGCTGATG; CXCL10 F: CTCTCTCTAGAACTGTACGCTG and CXCL10 R: ATTCAGACATCTCTTCTCACCC; CXCL11 F: GCTGTGATATTGTGTGCTACAG and CXCL11 R: TTGGGTACATTATGGAGGCTTT; T-bet F: CATTCCTGTCATTTACTGTGGC and T-bet R: CCCTTGTTGTTTGTGAGCTTTA; Tox F: GTTGACGTGAAGACATCTCAAC and Tox R: GACACAGCCATGTTTGCTATAG; Eomes F: ATTCATCCCATCAGATTGTCCC and Eomes R: ACGGTTCTCTCGCCATTATAAT; GAPDH F: ACAACTTTGGTATCGTGGAAGG and GAPDH R: GCCATCACGCCACAGTTTC. GAPDH served as a control to allow for normalization across samples so that the relative mRNA expression could be analyzed.

### Western blot analyses

Total protein was collected to examine protein phosphorylation status from A549 tumor cells and macrophages treated with supernatant or recombinant proteins for 2 h. Protein samples were obtained using pretreatment with ice-cold RIPA buffer containing a protease inhibitor cocktail (Thermo Flasher scientific) for 30 min. These protein samples were then subjected to electrophoresis and electrical transfer before incubation with primary antibodies against AKT (4691, Cell Signaling Technology, Massachusetts, USA), p-AKT (4060, Cell Signaling Technology, Massachusetts, USA), ERK (4695, Cell Signaling Technology, Massachusetts, USA), p-ERK (4370, Cell Signaling Technology, Massachusetts, USA), STAT3 (9139, Cell Signaling Technology), p-STAT3 (9145, Cell Signaling Technology, Massachusetts, USA), GAPDH (5174, Cell Signaling Technology, Massachusetts, USA). All western blot experiments were replicated at least three times.

### In vitro cytotoxicity assay

CIK cells were obtained from ex vivo expansion of PBMC for 14 days. CD4^-^CIK and CD4^+^ T cells were isolated using anti-human CD4 microbeads (Miltenyi Biotec, Germany) and co-cultured with A549, H520, or K562 cells for 4 h. Cytotoxicity assays were performed by measuring the optical density (OD) value from the release of lactate dehydrogenase (LDH) in the culture supernatant through the specific lysis of target cells.

### In vivo animal work

Female (NOD*/scid*–IL-2Rγ_c_^*null*^) NSG mice (5–6 weeks old) were purchased from SPF (Beijing) Biotechnology Co., Ltd. At day 0, the mice were subcutaneously injected with 5 × 10^6^ A549 cells/100 μL PBS. When the tumor growth was at around 100 mm^3^, the mice were randomly intravenously injected 1 × 10^7^ CIK, CD4^−^CIK, and CD4^+^ T cells/100 μL PBS. The tumor growth was measured using a vernier caliper three times a week. For T-cell tracer assay, 3 μM DIR (near-infrared dye) to label CIK cells to observe the movement of CD4^+^ T, CD4^-^CIK, and CIK cells through in vivo imaging at days 3, 5, 9, and 15. For migration assays in vivo, CXCR3 was blocked by intraperitoneal injection of AMG487 (a CXCR3 antagonist) every two days (5 mg/kg per mouse) to observe the tumor growth and T cells infiltration into the tumors. To examine the effect of rIL-17 and anti-PD-1 in inhibiting tumor growth in vivo, NSG mice were given different treatment regimens. For the rIL-17 + CD4^-^CIK group, intravenous injection of CD4^-^CIK cells (1 × 10^7^/mouse, once a week) was first performed through the tail vein, and rIL-17 (2 μg per mouse) injections were given the following day onwards, daily via i.p. for 4 days. For the anti-PD-1 + CD4^-^CIK group, i.p. injections of anti-PD-1 drug (150 μg/mouse, twice a week) was first performed, and intravenous injection of CD4^-^CIK cells (1 × 10^7^/mouse, once a week) was given the following day onwards. According to the previous study [26], the calculation formula of tumor volume is as follows: V = width × width × length/0.5. The mice were sacrificed by cervical dislocation at the endpoint of mice’s survival (mean tumor volume exceeded 1000 mm^3^ or tumor length diameter reached 1.5 cm). The fold change of tumor growth (FCTG) was calculated as follows: FCTG = (V_e_ − V_0_)/V_0_, where V_0_ is the tumor volumes measured at first treatment and V_e_ is the last tumor volumes measured in the tumor-bearing mice. For T-cell tracer assay and migration assays in vivo, a FCTG value <1 was recognized as effectively controlled tumor growth. For rIL-17 and anti-PD-1 experiments, FCTG value <2 was recognized as effectively controlled tumor growth.

### In vitro migration assays and co-culture

To investigate the transendothelial migration of CIK cells in vitro, a model containing upper and lower chambers (BD Falcon^TM^ HTS 3 μm FluoroBlok^TM^ 96-Multiwell Insert System, New Jersey, USA) was designed based on a previous study [14, 27]. HUVECs were grown for 2 days to achieve confluence in the upper wells. Tumor-conditioned medium were collected from the co-culture of tumor cells and macrophages (THP-1 cells was differentiated into macrophages using 100 ng/ml PMA) in the presence or absence of CD4^+^ T cells for two days (CD4^+^ T cells:A549 or H520 cells:macrophages = 5:1:1). 1 × 10^5^ CD4^-^CIK cells were seeded onto the upper well in the presence of the serum-free medium. The lower wells contained 150 μL tumor-conditional medium with either FCS, IFN-γ (40 ng/ml), anti-CXCR3 (500 ng/ml), or anti-IFN-γ (1 μg/ml). After transmigration for 24 or 48 h, CD4^-^CIK cells from the lower chambers were collected and counted using light microscopy or flow cytometry. In a co-culture model to assay the function of CIKs (CIKs or CD4^-^CIKs:A549 or H520 cells:macrophages = 5:1:1), CIK cells were collected for flow cytometry, after 3 days of co-culture with other cells. Tumor-conditioned medium, supplemented with either anti-IL-10 (200 ng/ml), anti-IL-17 (100 ng/ml), or Pi3K inhibitor (10 μM, Ly294002), were used to stimulate the CD4^-^CIK cells for 3 days.

### Enzyme-linked immunosorbent assay

The concentration of IL-21 was measured in the supernatant of tumor-conditioned medium by an ELISA kit according to the manufacturer’s protocol.

### In vitro Th17 differentiation

PBMCs were first extracted from normal blood. Purified CD4^+^ T cells were then obtained from PBMC using anti-CD4 microbeads (Miltenyi Biotec, Germany) according to the manufacturer’s protocol. The purified CD4^+^ T cells were stimulated and activated with anti-CD3/CD28 (25 μg/ml) antibody complexes in basal medium for three days. To promote T-cell differentiation into Th17, IL-6 (30 ng/mL), IL-1β (30 ng/mL), IL-23 (30 ng/mL), and TGFβ (2.25 ng/mL) were added to the basal medium according to a previous study [28].

### Tumor tissue preparation and flow cytometry

Spleen was prepared into single-cell suspension by mechanical grinding. Liver and tumor tissues were prepared into single-cell suspension by enzymatic digestion (collagenase IV 1 mg/ml and Dnase I 50 μg/ml). All single-cell suspension was passed through a 70-μm cell strainer (BD Falcon, New Jersey, USA) to remove impurities before erythrocyte lysis was performed. For intracellular staining of GzmB and IFN-γ, the cells were treated with leukocyte activation cocktail with BD GolgiPlug (BD Biosciences, New Jersey, USA) in vitro. Cell surface antigen staining was performed before the cells were fixed and permeabilized with the BD Cytofix/Cytoperm kit (BD Biosciences, New Jersey, USA) for subsequent intracellular staining. Flow cytometry data were analyzed with FlowJo software. Live/Death cells were selected/excluded based on LIVE/DEAD™ Fixable Dead Cell Stain Kits (Invitrogen™, California, USA).

### Immunohistochemical staining

Tumor tissues of transplanted NSG mice were prepared into paraffin sections (4 μm). After deparaffinization, rehydration, and antigen retrieval, the tissues were probed with anti-CD8 primary antibody (dilution: 1:10,000, Proteintech, China) overnight. EIVISON plus (kit-9903, MXB, China), DAB kit (ZL1-9019, ZSGB-BIO, China) were used for coloration the following day, according to the manufacturer’s protocol. Stained tumor sections were evaluated under a light-field microscope (Olympus, Japan).

### Statistical analyses

All clinical statistical analyses were performed using SPSS v.21 (IBM Corp, New York State, USA), and all statistical graphs and survival curves were drawn using GraphPad Prism 8 (GraphPad Software, USA). OS and PFS were calculated by Kaplan–Meier survival analysis. One-way ANOVA with Dunn’s multiple comparisons was performed for tumor growth analysis and comparisons between three or more groups. Differences between two groups were analyzed using unpaired Student’s *t* test. *P* < 0.05 was considered statistically significant.

## Discussion

CIK cell therapy, an adoptive treatment method, has shown promising results in multiple types of cancers, especially in hematological tumors. However, it still has little effect in the treatment of solid tumors. In addition, CIKs are a heterogeneous cell group, and the mutual regulation mechanism and potential relationship among the various cell subgroups remains unclear. Herein, through analysis of the one clinical trial data, the different percentages of CD4^+^ T, Treg, and CD3^+^CD56^+^ T cells in CIKs derived from ex vivo expansion were found to ultimately affect the therapeutic efficacy. These findings also suggest that CD4^+^ T cells play an important role in CIK therapy.

Previous study has demonstrated that the CD4^+^ T cells can positively regulate the function of CTL by enhancement of CTL activity, migratory, and survival at tumor site [8]. However, some CD4^+^ Th cells, such as Treg cells, can also suppress anti-tumor immune response; they strongly exert immune suppression by inducing CTL exhaustion in tumor microenvironment [29]. Herein, CD4^+^ T cells, especially Th1/Th17 cell subsets, were found to inhibit the expression of inhibitory receptors on CIKs, enhance the tumor-killing function of CIKs, and promote the migration and locomotion of CIKs. This phenomenon was not only reflected as anti-tumor responses, but even in the early stage of CIK cells in vitro expansion. Among them, the cytokines of IFN-γ and IL-17A improved the function of CIK cells particularly and significantly.

At present, however, the roles of IL-17A and Th17 cell subsets in the tumor microenvironment are controversial. Some studies have revealed a strong tumor-killing effect in the adoptive transfer of Th17 and Th9 [30]. Other studies have also shown that IL-17A can activate NK cells, as evidenced by upregulated CD107a, TNF-α, IFN-γ, and NKG2D expression in NK cells, or induce IL-6 production from macrophages to promote the survival, recruitment, and cytotoxicity of CTLs [31, 32]. However, several studies have demonstrated that Th17 settled inside tumors can accelerate tumor cell metastasis or reduce the migration ability of CD8^+^ T cells, and promote the ability of tumor cells to evade immune attack [33–35]. Thus, the role of IL-17A needs to be re-recognized in coordination with changes in the tumor immune microenvironment.

Previous studies have demonstrated PI3K/AKT pathway as an important pathway for the activation and promotion of cytokine production by various immune cells, including NK cells and CTLs [36, 37]. Meanwhile, T-bet and Eomes as the important transcription factors were regulated by PI3K/AKT pathway and affected the cytokine production (such as IFN-γ and GzmB) of NK cells, especially T-bet expression [19, 38]. Herein, rIL-17A was found to improve the function and restore the dysfunction of CD4^-^CIK cells by inducing the phosphorylation of the AKT pathway and up-regulating the expression of T-bet and Eomes.

In conclusion, the present study revealed that the Th1/Th17 cell subsets in the CIK cell population can effectively improve the function of T cells and recruit T-cell infiltration into tumor tissues by releasing IL-17A and IFN-γ. In addition, CD4^+^ T cells were also demonstrated to effectively reverse the functional exhaustion of CIK cells infiltration into NSCLC and restore the cytotoxicity of CIK cells through the IL-17/AKT/T-bet axis. Furthermore, CIK therapy plus anti-PD-1 treatment was shown to be a feasible combination therapy method to promote the anti-tumor response of CIK cells and inhibit tumor progression, especially for NSCLC patients with dysfunctional and low proportion of CD4^+^ T cells in their peripheral blood. Therefore, these findings provide an experimental and theoretical basis for the clinical screening of suitable patients for CIK treatment, and also propose a new combination therapy method for CIK treatment application in solid tumors, including NSCLC.

## Supplementary information

Supplementary information

Original Data File

reproducibility checklist

## Data Availability

The data used and analyzed during this study are available from the corresponding author on reasonable request.
