# Supplementary material for: CD4+ T cells are required to improve the efficacy of CIK therapy in non-small cell lung cancer
Source: Cell Death Dis. 2022 May 6;13(5):441. doi: 10.1038/s41419-022-04882-x (PMC9076680; doi:10.1038/s41419-022-04882-x)
Supplement: Supplementary file 1 — Supplementary information [file 41419_2022_4882_MOESM1_ESM.doc]

**Supplementary information includes 6 figures and 2 tables.**

**Table S1: Clinical characteristics of 43 patients with advanced LUSC.**

| **Characteristics** |  | **ALL(n)** |
| --- | --- | --- |
| Gender | Male | 33 |
|  | Female | 10 |
| Age | > 60 | 16 |
|  | ≤ 60 | 27 |
| Smoke | No | 10 |
|  | Yes | 33 |
| T stage | T1 | 3 |
|  | T2 | 12 |
|  | T3 | 9 |
|  | T4 | 19 |
| N stage | N0 | 4 |
|  | N1 | 1 |
|  | N2 | 20 |
|  | N3 | 18 |
| M stage | M0 | 15 |
|  | M1a | 14 |
|  | M1b | 14 |
| Clinical stage | IIIB | 15 |
|  | IV | 28 |

**Table S2: Univariate and** **multivariate cox analysis of factors for PFS and OS in patients with LUSC.**

| **Variable** | **Progression-free survival** | | | **Overall survival** | | |
| --- | --- | --- | --- | --- | --- | --- |
| **Multivariate cox analysis** | **HR** | **95%CI** | ***p*-value** | **HR** | **95%CI** | ***p*-value** |
| T factor (T≤ 2 vs.T > 2) | 0.638 | 0.291-1.4 | 0.262 | 0.731 | 0.252-2.123 | 0.564 |
| N factor (N ≤ 2 vs. N > 2 ) | 0.936 | 0.416-2.109 | 0.874 | 0.36 | 0.12-1.081 | 0.069 |
| M factor (M0 vs. M1) | 0.497 | 0.214-1.15 | 0.102 | 1 | 0.348-2.873 | 0.999 |
| Percentage of CD4^+^ T cells |  |  | **0.026** |  |  | 0.432 |
| Low group vs. high group | 0.445 | 0.159-1.244 | 0.123 | 2.314 | 0.65-8.24 | 0.196 |
| Intermediate group vs. high group | 0.198 | 0.061-0.643 | **0.007** | 1.712 | 0.497-5.897 | 0.394 |
| Percentage of CD3^+^CD56^+^ T cells (Low vs. high) | 1.526 | 0.631-3.691 | 0.348 | 4.466 | 1.487-13.411 | **0.008** |
| Percentage of Treg (Low vs. high) | 0.388 | 0.165-0.914 | **0.03** | 0.123 | 0.034-0.445 | **0.001** |

Statistically significant *p*-values (*p* < 0.05) were shown in **Bold**

**
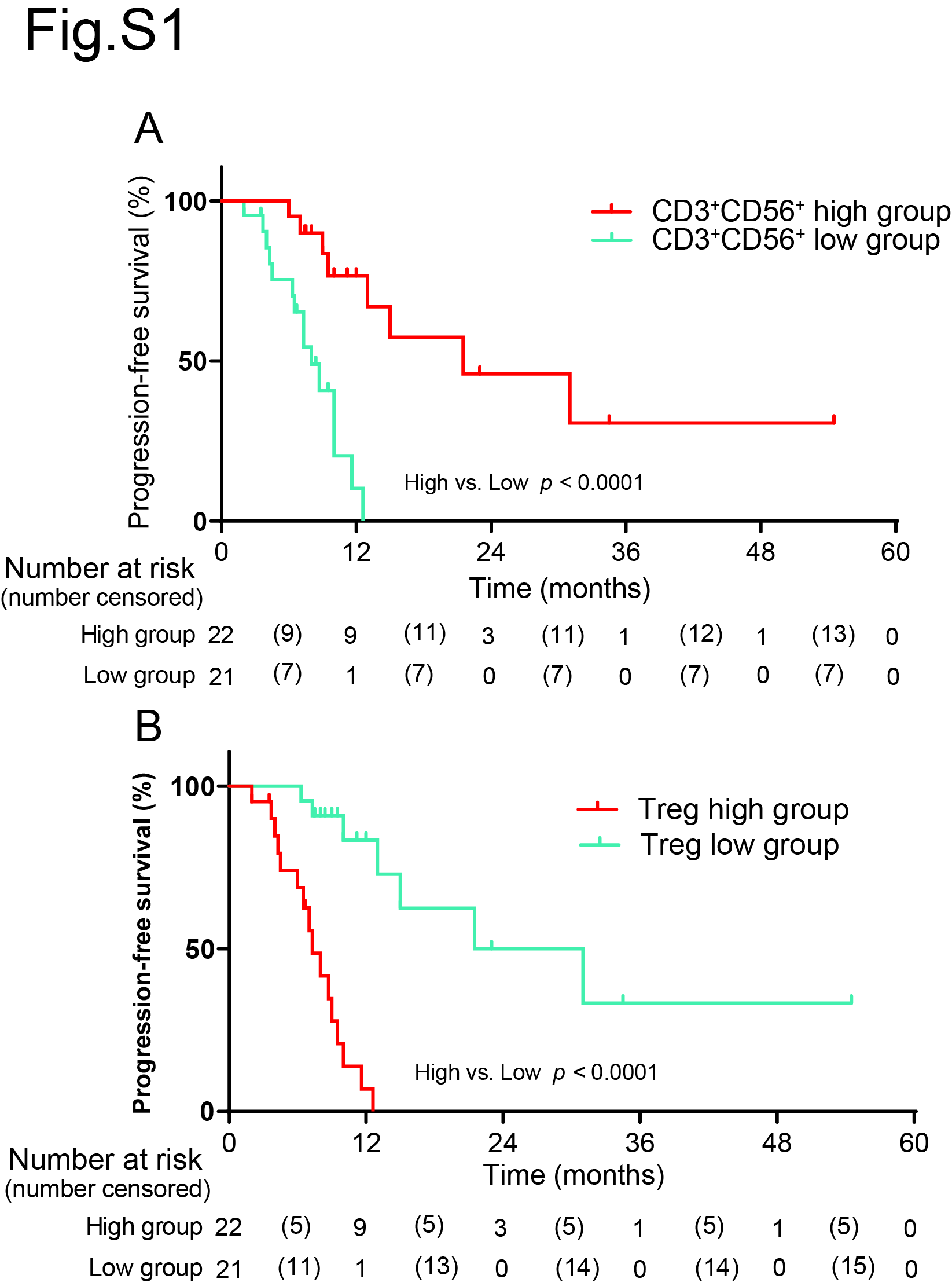
**

**Figure S1: The percentage of CD3^+^CD56^+^ T cells and Treg cells correlated with clinical prognosis. (A)** Kaplan-Meier estimates of progression-free survival in the two groups (CD3^+^CD56^+^ high group and low group). **(B)** Kaplan-Meier estimates of progression-free survival in the two groups (Treg high group and low group).*p < 0.05, **p < 0.01, and ***p < 0.001.

**
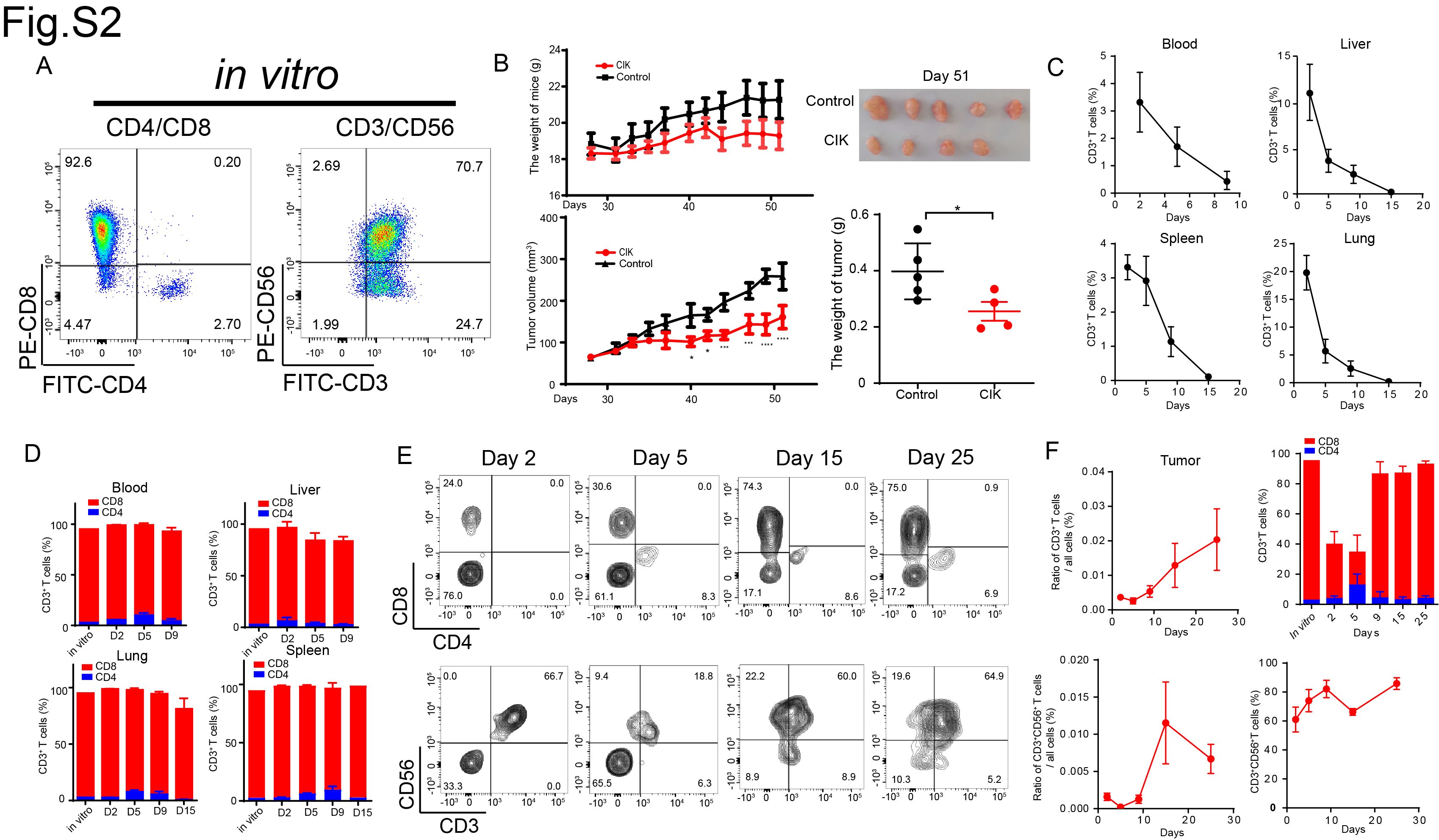
**

**Figure S2: The number of CIKs were gradually increased over time at tumor sites.** **(A)** Flow cytometric quality assessment of CIKs after 14 days of *ex-vivo* expansion before adoptive to tumor-burdened NSG mice. **(B)** Left: Subcutaneous growth of tumor cells (A549) and the weight of mice in each group treated with CIK cells (n = 4); right: Specific images and the weight of subcutaneous transplanted tumors from two groups. **(C)** Flow cytometric analysis of the percentage of CD3^+^ T cells in spleen, blood, liver, and lung after CIKs were injected to mice. **(D)** Flow cytometric examination of CD4:CD8 ratio in spleen, blood, liver, and lung after CIKs were injected to mice. **(E)** Specific imaging of CD3^+^CD8^+^ T cells and CD3^+^CD56^+^ T cells in tumor tissues from flow cytometry after CIKs were injected to mice. **(F)** Flow cytometric examination of the percentage of CD3^+^ T and CD3^+^CD56^+^ T cells in tumor sites after CIKs were injected to mice. Error bars indicate SEM, *p < 0.05, **p < 0.01, ***p < 0.001, and ****p < 0.0001.

**
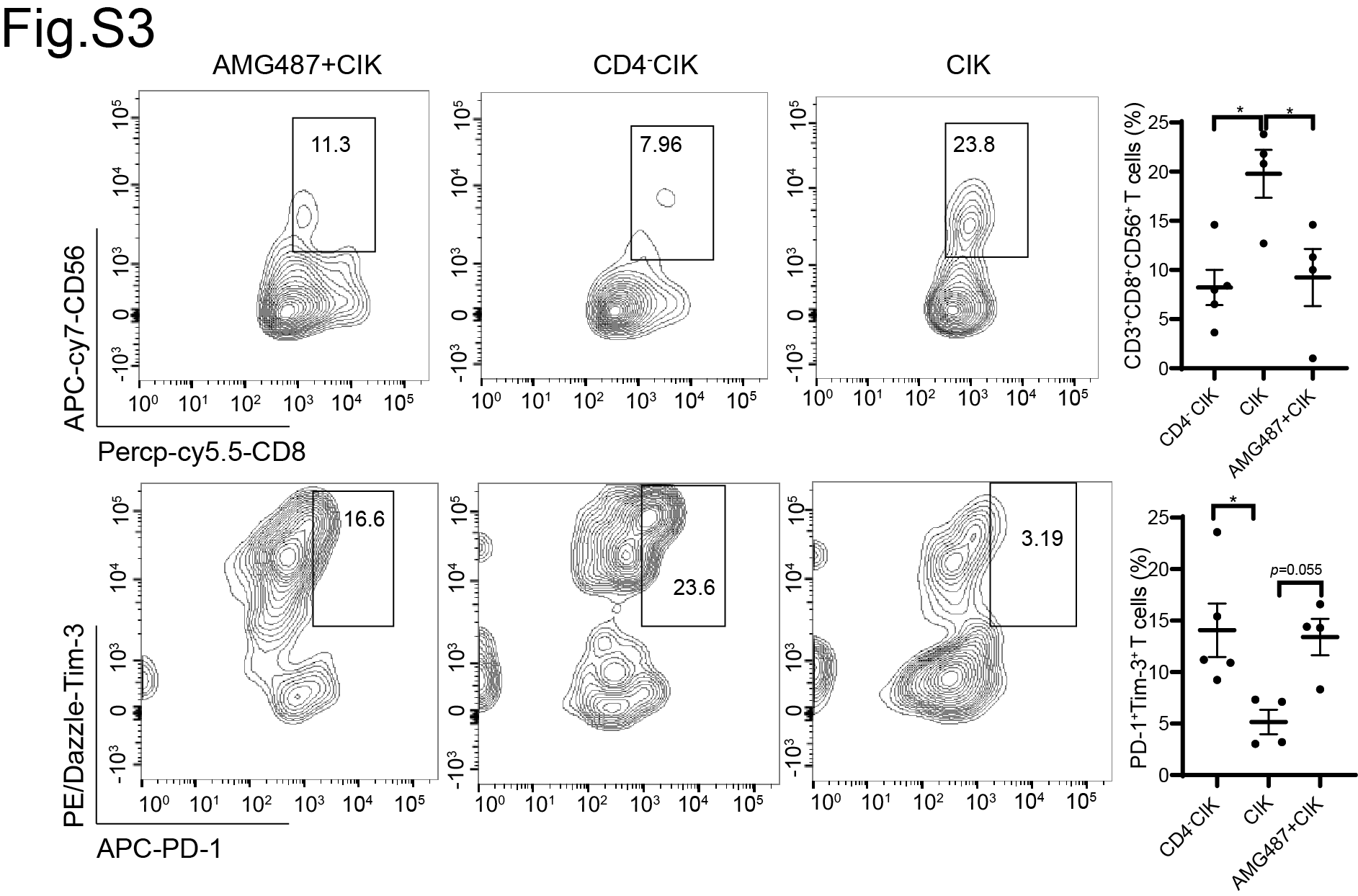
Figure S3: AMG487 inhibited the infiltration of CD3^+^CD56^+^ T cells**. Subcutaneous growth of tumor cells (A549) in each group of mice (n = 5) treated with PBS, CD4^-^CIK cells, CIK cells, and AMG487+CIK. Flow cytometric examination of the percentage of CD3^+^CD8^+^CD56^+^ T and PD-1^+^Tim-3^+^ T cell infiltration into tumor. Error bars indicate SEM, *p < 0.05, **p < 0.01, and ***p < 0.001.

**
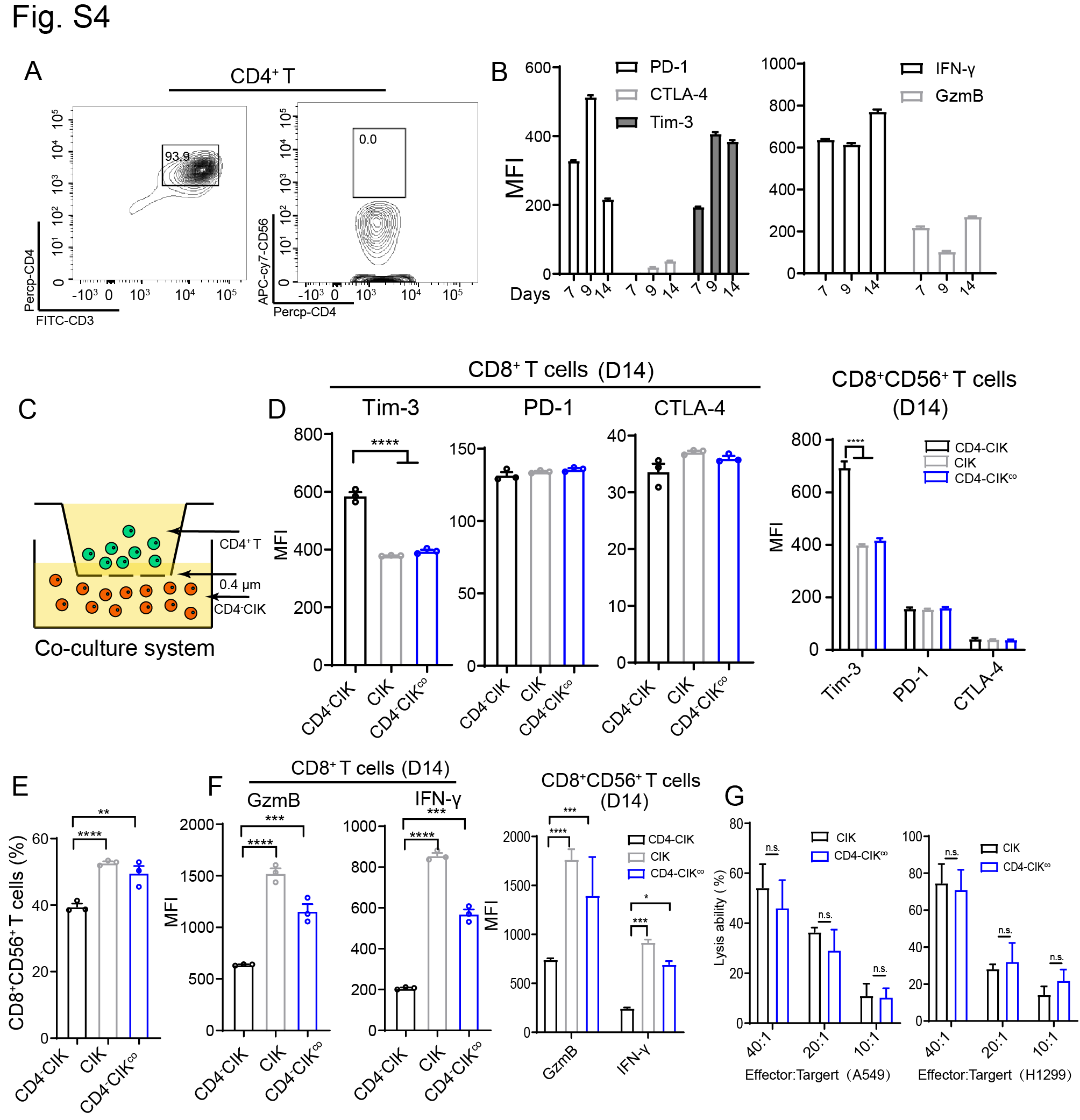
Figure S4:** **CD4^+^ T cells enhanced the function of CIKs independent of intercellular contacts during *ex-vivo* expansion**. **(A)** Flow cytometric quality assessment of CD4^+^ T cells when *ex-vivo* expansion at day 14. **(B)** Flow cytometric examination of immune checkpoint receptors expression, IFN-γ and GzmB expression in CD3^+^CD4^+^ T cells. **(C)** Schematic diagram depicting the *ex-vivo* expansion of CD4^-^CIKs in co-culture system (CD4^-^CIK^CO^). **(D)** Flow cytometric examination of immune checkpoint receptors expression in CD3^+^CD8^+^ T cells and CD3^+^CD8^+^CD56^+^ T cells. **(E)** Flow cytometric examination of the percentage of CD3^+^CD8^+^CD56^+^ T cells in CD3^+^CD8^+^ T cells. **(F)** Flow cytometric analysis of IFN-γ and GzmB production in CD3^+^CD8^+^ T cells and CD3^+^CD8^+^CD56^+^ T cells. **(G)** The cytotoxicity of CIK or CD4^-^CIK^CO^ cells against A549 and H1299. Error bars indicate SEM, *p < 0.05, **p < 0.01, ****p < 0.001, and ****p < 0.0001 (one-way ANOVA or Students’t test).

**
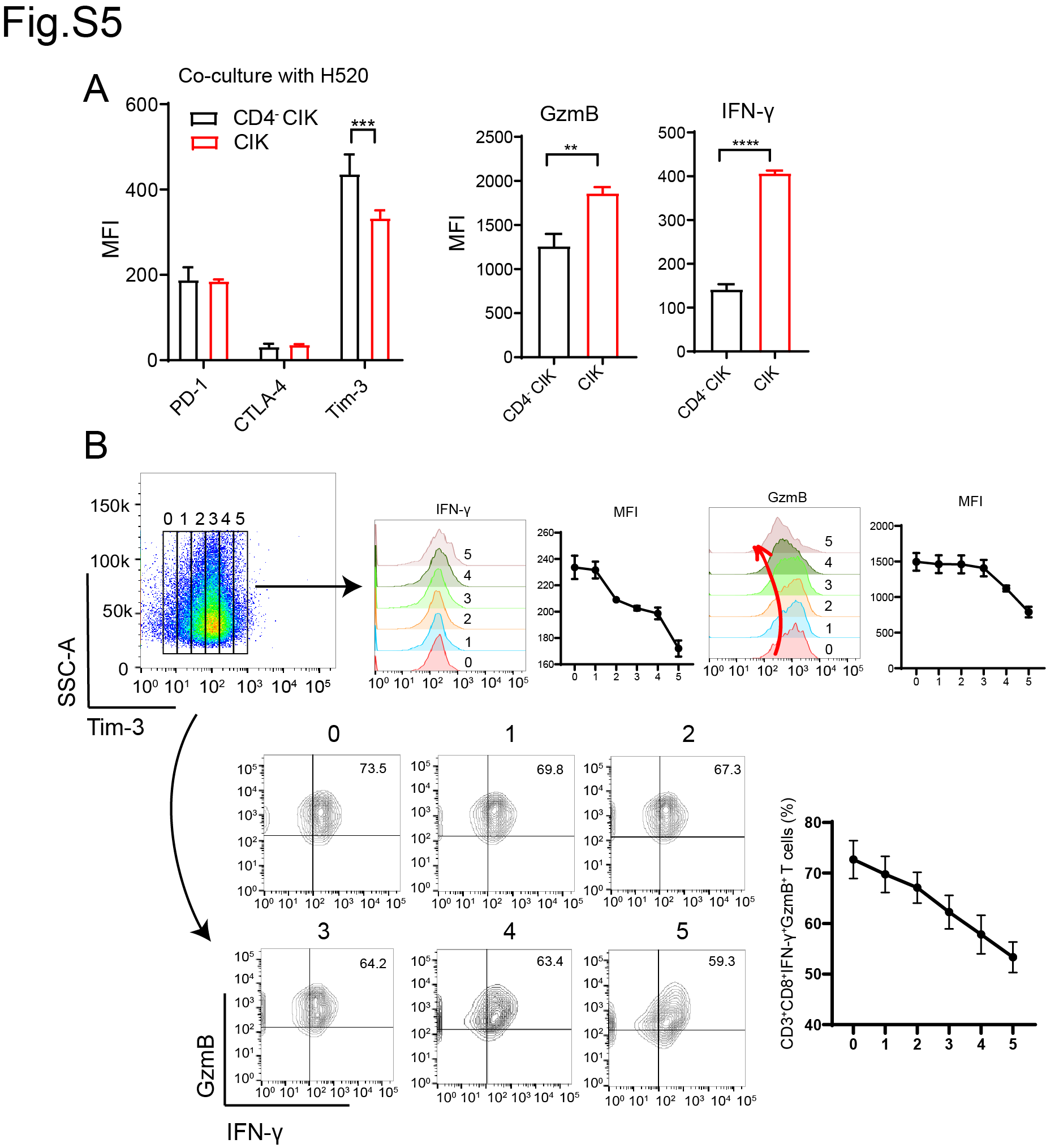
**

**Figure S5: Tim-3 expression was closely related to the function of CIKs**. **(A)** Flow cytometric analysis of PD-1, CTLA-4, Tim-3, GzmB and IFN-γ expression in CD3^+^CD8^+^ T cells. **(B)** CD8^+^ T cells in the co-culture system were equally divided into six groups (0, 1, 2, 3, 4, and 5) based on the level of Tim-3 expression and flow cytometric analysis of IFN-γ and GzmB expression in CD3^+^CD8^+^ T cells. Error bars indicate SEM. *p < 0.05, **p < 0.01, ***p < 0.001 and ****p < 0.0001 (one-way ANOVA or Students’t test).

**
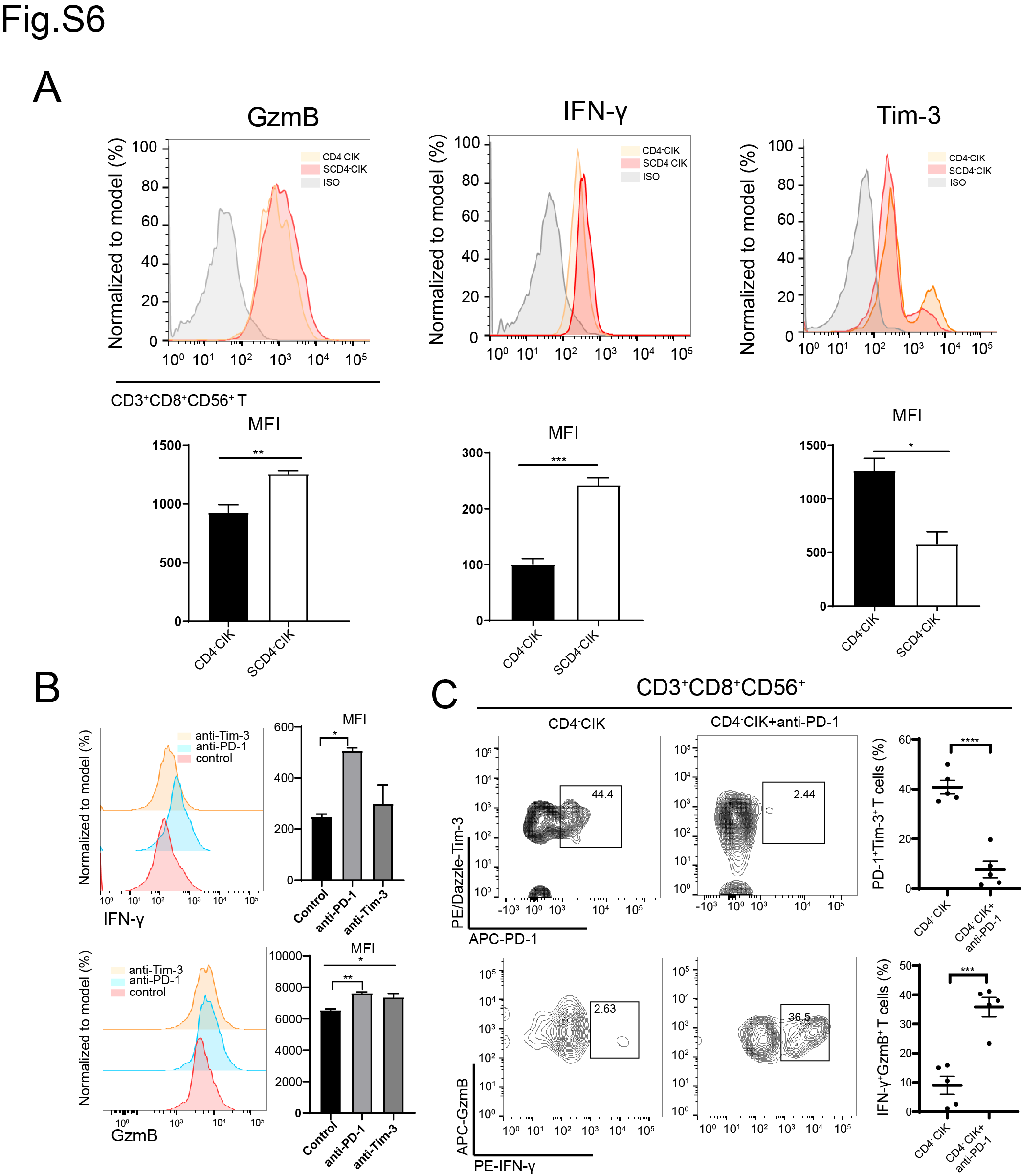
**

**Figure S6 : CIK cell therapy plus anti-PD-1 treatment reversed the functions of exhausted T cells*.* (A)** Flow cytometric analysis of Tim-3, IFN-γ and GzmB expression were examined in CD3^+^CD8^+^CD56^+^ T cells. **(B)** Flow cytometric analysis of IFN-γ and GzmB expression were examined in CD3^+^CD8^+^ T cells treated with anti-PD-1 Abs or anti-Tim-3 Abs. Subcutaneous growth of tumor cells (A549) in each group of mice treated with CD4^-^CIK cells and CD4^-^CIK cells+anti-PD-1. **(C)** Flow cytometric analysis of the percentage of PD-1^+^Tim-3^+^ T cells and GzmB^+^IFN-γ^+^ T cells were examined in CD3^+^CD8^+^CD56^+^ T cells. Error bars indicate SEM, *p < 0.05, **p < 0.01, ***p < 0.001 and ****p < 0.0001 (one-way ANOVA or Students’t test).
