# Supplementary figures and images for: CD4+ T cells are required to improve the efficacy of CIK therapy in non-small cell lung cancer
Source: Cell Death Dis. 2022 May 6;13(5):441. doi: 10.1038/s41419-022-04882-x (PMC9076680; doi:10.1038/s41419-022-04882-x)

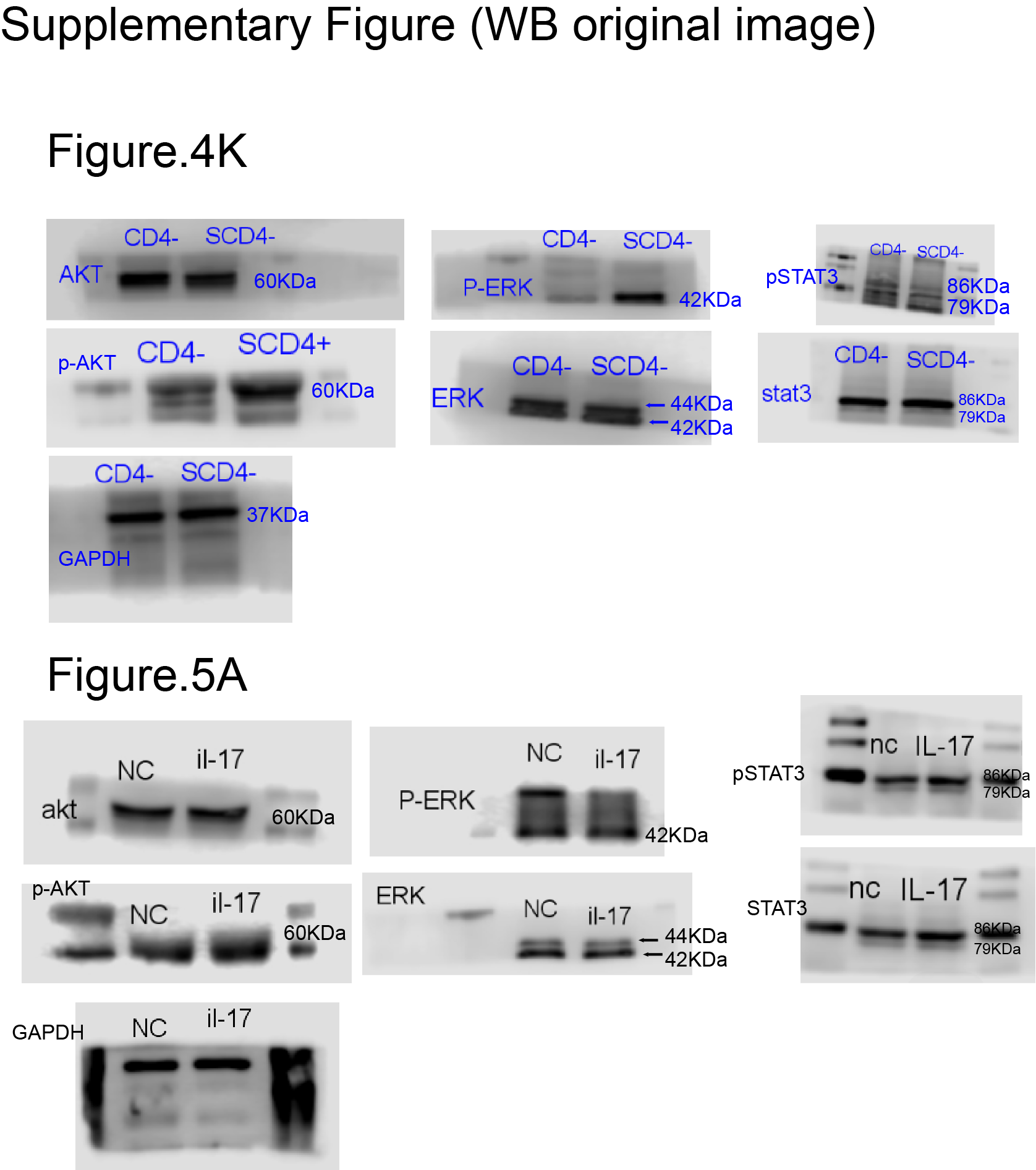

Supplement: Supplementary file 2 — Original Data File [file 41419_2022_4882_MOESM2_ESM.png]
